# Assessment of Accuracy of Mixed Reality Device for Neuronavigation: Proposed Methodology and Results

**DOI:** 10.1227/neuprac.0000000000000036

**Published:** 2023-04-14

**Authors:** Swati Jain, Tamara Tajsic, Tilak Das, Yujia Gao, Ngiam Kee Yuan, Tseng Tsai Yeo, Martin J. Graves, Adel Helmy

**Affiliations:** * Divison of Neurosurgery, University Surgical Cluster, National University Health System, Singapore;; ‡ Division of Neurosurgery, Department of Clinical Neurosciences, University of Cambridge, Cambridge, UK;; ‖ Division of Hepatobiliary & Pancreatic Surgery, University Surgical Cluster, National University Health System (NUHS), Singapore;; ¶ Division of General Surgery (Thyroid & Endocrine Surgery), University Surgical Cluster, National University Health System (NUHS), Singapore;; § Department of Radiology, Cambridge University Hospitals NHS Foundation Trust, Cambridge, UK

**Keywords:** HoloLens 2, Mixed reality, Neuronavigation

## Abstract

Intraoperative neuronavigation is currently an essential component of neurosurgical operations in several contexts. Recent progress in mixed reality (MR) technology has attempted to overcome the disadvantages of standard neuronavigation systems allowing the surgeon to superimpose a 3D rendered image onto the patient's anatomy. We present the first study in the literature to assess the surface matching accuracy of MR rendered image. For the purposes of this study, we used HoloLens 2 with virtual surgery intelligence providing the software capability for image rendering. To assess the accuracy of using mixed reality device for neuronavigation intraoperatively. This study seeks to assess the accuracy of rendered holographic images from a mixed reality device as a means for neuronavigation intraoperatively. We used the Realistic Operative Workstation for Educating Neurosurgical Apprentices to represent a patient's skull with intracranial components which underwent standardized computed tomography (CT) and MRI imaging. Eleven predefined points were used for purposes of assessing the accuracy of the rendered image, compared with the intraoperative gold standard neuronavigation. The mean HoloLens values against the ground truth were significantly higher when compared with Stealth using CT scan as the imaging modality. Using extracranial anatomic landmarks, the HoloLens error values continued to be significantly higher in magnitude when compared with Stealth across CT and MRI. This study provides a relatively easy and feasible method to assess accuracy of MR-based navigation without requiring any additions to the established imaging protocols. We failed to show the equivalence of MR-based navigation over the current neuronavigation systems.

**ABBREVIATIONS:**
GT: ground truth
HL: HoloLens point
MR: mixed reality
ROWENA: Realistic Operative Workstation for Educating Neurosurgical Apprentices.

Intraoperative neuronavigation is currently an essential component of many neurosurgical operations. Preoperative surgical planning, intraoperative localization of deep-seated tumors, stereotactic biopsies, and determining intraoperative resection margins for intrinsic tumors are some of the common applications of the intraoperative navigation. Advancements in imaging have furthered its usage such as planning minimally invasive surgeries and mapping white matter tracts.

However, there are several disadvantages that are associated with currently available systems.^1-3^ First, there is only 2D projection of images on the screen requiring the surgeon to mentally reconstruct this into a 3D image during planning. While some systems have incorporated the 3D reconstruction, it is rigid based on the preoperative planning of surgery. Second, any errors in navigation after preparation and draping require re-registration and restarting of the entire process. Errors of few millimeters may result in missing the target lesions especially in stereotactic biopsies or lead to injury of critical neurovascular structures. Finally, intraoperative instruments or their paths of insertion cannot be visualized unless they have been preregistered with the navigation system used.

Recent progress in mixed reality (MR) technology has attempted to overcome the disadvantages of standard neuronavigation systems allowing the surgeon to superimpose a 3D rendered image onto the patient's anatomy. Conventionally, MR has been mainly used in education. A few studies have assessed the utility of MR as an adjunct to conventional neuronavigation in procedures such as external ventricular drain placement and spinal pedicle screws insertion.^4-6^ However, no studies have assessed the surface matching accuracy of the MR rendered image. The accuracy with which the image is rendered will directly affect the downstream usage of the MR for various surgical procedures.

We present the first study in the literature to assess the surface matching accuracy of MR rendered image. For the purposes of this study, we used HoloLens 2 (Microsoft HoloLens 2) with Virtual Surgery Intelligence (VSI HoloMedicine by apoQlar) providing the software capability for image rendering. We used Realistic Operative Workstation for Educating Neurosurgical Apprentices (ROWENA, Delta Surgical, and NeuroSurgical Ltd),^7^ a durable head base, featuring face, neck, and ears, and with accurate internal skull base anatomy. Comparisons were made against a widely used commercial neuronavigation platform and against defined anatomic landmarks on the ROWENA model.

## METHODS

### Preoperative Imaging

For the purposes of this study, we used the ROWENA model to represent a patient's skull with intracranial components. The model was scanned using computed tomography (CT) and MRI using pre-established standard neuronavigation protocols. These images were uploaded to the HoloLens for 3D rendering and evaluation. The images were uploaded to the StealthStation (StealthStation S8 system, Medtronic) for registration for neuronavigation. For the purposes of this study, noncontrast CT and T1-weighted MRI images were used.

### Positioning and Registration

**FIGURE 1.**
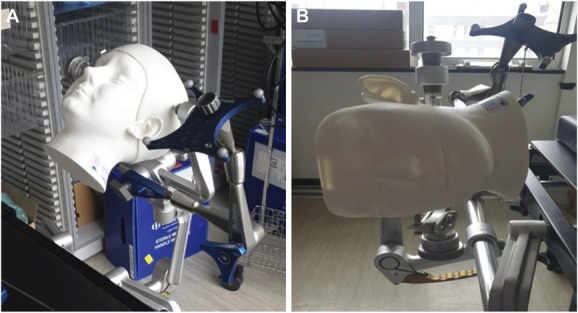
**A**, This figure shows the ROWENA head clamped in a supine position. **B**, This figure shows the ROWENA head clamped in a supine position. ROWENA, Realistic Operative Workstation for Educating Neurosurgical Apprentices.

A ROWENA skull was clamped using a Mayfield clamp attached to a standard operating table (Figure 1A and 1B). The skull cover and intracranial components were removed as bony landmarks were used for the assessment of accuracy. Two positions were used for the purposes of this study—neutral (Figure 1A) and left lateral position (Figure 1B). Optical tracking for tumor resection module in Stealth S8 was used for registration. The registration accuracy was recorded for each imaging modality in the 2 different positions. The 3D rendered image was projected onto ROWENA using HoloLens 2. Video is available in the supplemental content. It shows the superimposed hologram with the ROWENA in the supine position.

**VIDEO.** The video shows the superimposed hologram with the ROWENA in the supine position.

### Assessment of Accuracy

Eleven predefined points were used for the purposes of this study—(1) right medial canthus, (2) left medial canthus, (3) right lateral canthus, (4) left lateral canthus, (5) tip of the nose, (6) right ear tragus, (7) left ear tragus, (8) right anterior clinioid, (9) left anterior clinoid, (10) right internal auditory meatus, and (11) left internal auditory meatus. Stealth surgical plans were used to measure the deviations among the modalities. The 3 plans used are as follows:

Plan 1: Target set as ground truth (GT) and entry set as Stealth point (S), where GT was defined based on the landmark under direct visualization and S defined as the landmark based on Stealth visualization.

Plan 2: Target set as GT and entry set as HoloLens point, where the HoloLens point was defined based on the landmark based on HoloLens visualization.

Plan 3: Target set as GT and entry set as Stealth point (S).

**FIGURE 2.**
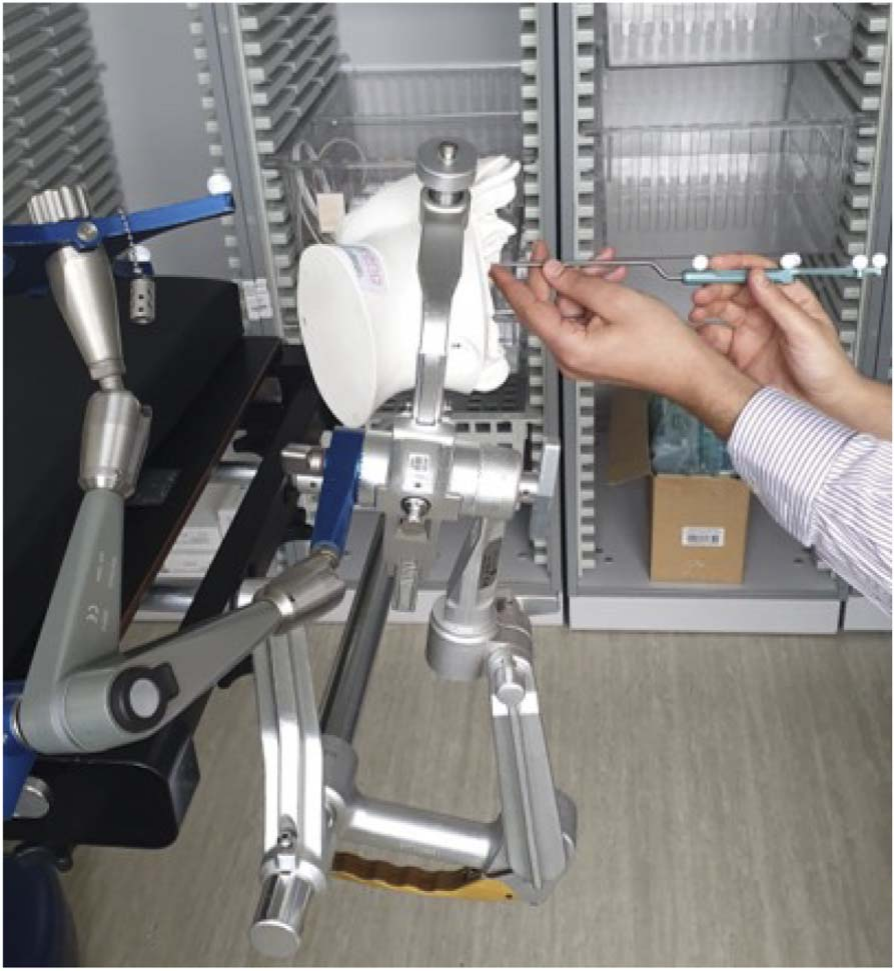
This figure shows one of the operators using the Stealth probe to register the ground truth.

We used a 5-mm threshold as an acceptable margin of error.^3,8-10^ Three independent operators were involved in performing this study. Two operators interchanged using the HoloLens and StealthStation for both modalities in neutral and supine position. Each operator was blinded to the results of the assessment. Figure 2 shows one of the operators using the Stealth probe to register the GT.

All statistical tests were performed using STATA 17 (STATA ® 17, StataCorp LLC). Paired Student *t*-tests were used to compare across the various measurements for each anatomic location.

## RESULTS

**FIGURE 3.**
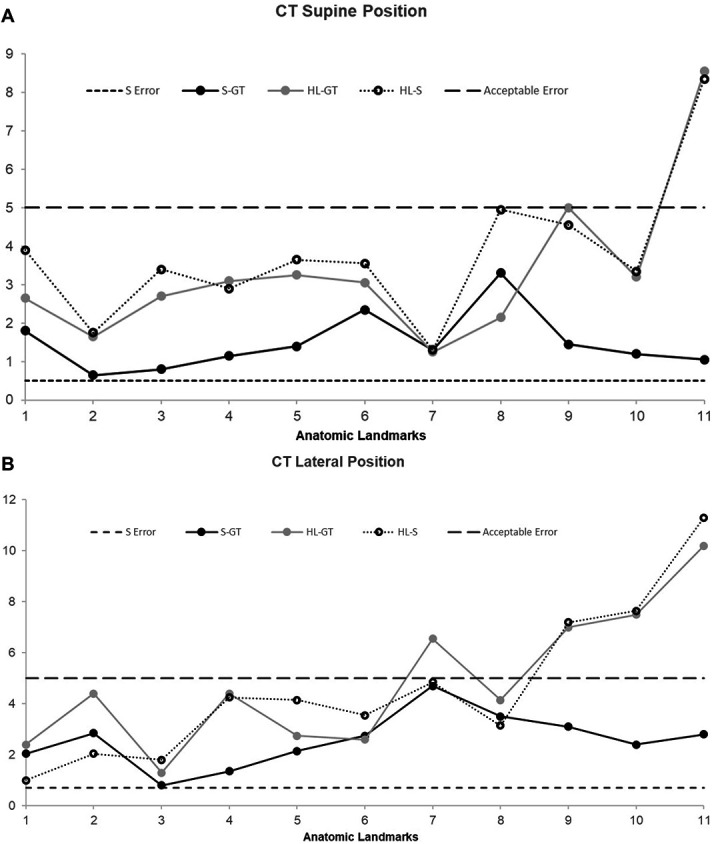
**A**, This figure presents the scatter plot results of the magnitude of error when compared with the ground truth using CT as imaging modality with the model in supine position. **B**, This figure presents the scatter plot results of the magnitude of error when compared with the ground truth using CT as imaging modality with the model in lateral position. CT, computed tomography.

**FIGURE 4.**
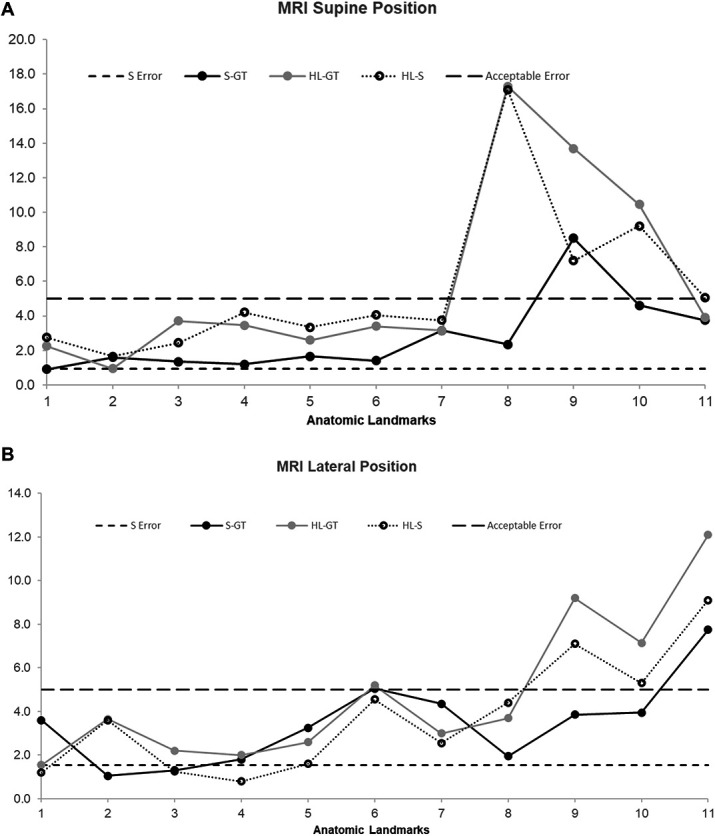
**A**, This figure presents the scatter plot results of the magnitude of error when compared with the ground truth using MRI as imaging modality with the model in supine position. **B**, This figure presents the scatter plot results of the magnitude of error when compared with the ground truth using MRI as imaging modality with the model in lateral position. GT, ground truth; HL, HoloLens point.

Figures 3 and 4 show the scatter plot results of the CT and MRI in supine and lateral position, respectively. All measurements were plotted as a mean of the 2 readings obtained by the 2 independent operators. For the purposes of this study, 5 mm was used as the acceptable accuracy level. Both extracranial and intracranial landmarks were used for the purposes of this study as described in materials and methods.

**FIGURE 5.**
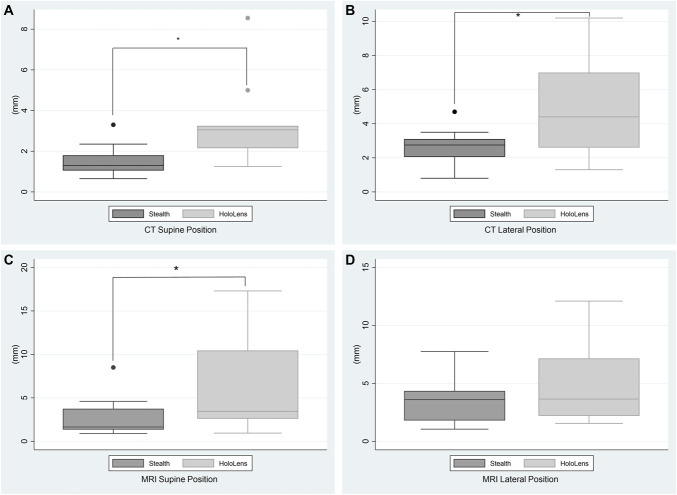
This figure shows the box and whisker plot for the 4 events. **A**, This figure shows the comparison between the magnitude of the errors from ground truth between Stealth and HoloLens using CT as imaging modality in a supine position. **B**, This figure shows the comparison between the magnitude of the errors from ground truth between Stealth and HoloLens using CT as imaging modality in a lateral position. **C**, This figure shows the comparison between the magnitude of the errors from ground truth between Stealth and HoloLens using MRI as imaging modality in a supine position. **D**, This figure shows the comparison between the magnitude of the errors from ground truth between Stealth and HoloLens using MRI as imaging modality in a lateral position. CT, computed tomography.

Table presents the various parameters across the 2 imaging modalities in supine and lateral position. Figure 5 shows the box and whisker plot for the 4 events. The mean HoloLens values against the ground truth were significantly higher when compared with Stealth using CT scan as the imaging modality. Although the median and mean continued to be lower than the acceptable accuracy of 5 mm, it still did not reach the accuracy level provided by the gold standard form of neuronavigation. The results remained similar when the 2 modalities were compared using MR in supine position as shown in Figure 5C. Interestingly, when the 2 modalities were compared using MR in lateral position, there was no significant difference between HoloLens and Stealth (Figure 5D).

**TABLE.** Magnitude of Errors Across the 2 Imaging Modalities in Supine and Lateral Position, Divided by Intracranial and Extracranial Landmarks

	Extracranial landmarks						
	n	Supine			Lateral		
		Stealth	HoloLens	*P* value	Stealth	HoloLens	*P* value
CT							
Mean	7	1.35	2.52	.0064	2.78	3.48	.0378
SD (±)		0.58	0.77		1.25	1.75	
Median		1.3	2.7		2.15	2.75	
MRI							
Mean	7	1.6	2.78	.0356	2.91	2.88	.9622
SD (±)		0.72	0.96		1.55	1.22	
Median		1.4	3.15		3.25	2.6	

	Intracranial landmarks						
	n	Supine			Lateral		
		Stealth	HoloLens	*P* value	Stealth	HoloLens	*P* value
CT							
Mean	4	1.75	4.72	.1965	2.95	7.21	.0563
SD (±)		1.04	2.81		0.46	2.47	
Median		1.32	4.1		2.95	7.25	
MRI							
Mean	4	4.8	11.33	.1239	4.37	8.03	.0179
SD (±)		2.64	5.69		2.43	3.53	
Median		4.17	12.07		3.9	8.17	

CT, computed tomography.

The data sets were subsequently divided into intracranial and extracranial anatomic landmarks. Table presents the various parameters across the 2 imaging modalities separated by intracranial and extracranial components. Using extracranial anatomic landmarks, the HoloLens error values continued to be significantly higher in magnitude when compared with Stealth across CT in both positions and MRI in supine positions. This replicates the results as obtained earlier when both extracranial and intracranial landmarks were combined.

The statistical significance between HoloLens and Medtronic S8 was lost when only the intracranial landmarks were compared. The HoloLens deviated with much higher values from the acceptable accuracy of 5 mm as presented in Table. Stealth continued to remain below the acceptable accuracy within the intracranial anatomic landmarks.

## DISCUSSION

Mixed reality technology has opened new avenues for planning, visualization, and education in surgery. It eliminates the need for mental image processing from a displayed 2D to the 3D anatomy in the operating room, and it does not require the use to stop and review the neuronavigation image on a distantly displayed screen while operating. While the 3D rendered images are attractive, their safety and efficacy, interuser variability, and application in the operating theater remain unassessed. A systemic review by Cho et al^11^ revealed nearly 26 cases of intracranial neurosurgical cases where virtual reality or augmented reality was used. The first-generation HoloLens was shown to have higher registration and planning time when used in 25 patients. This same system was found to be clinically useful in 6 patients in localizing superficial tumors and optimizing incision planning and craniotomy.^12^

Despite an increase in the number of publications on usage of mixed reality in operating theater, there remains a lack of evidence on its accuracy when compared with gold standard neuronavigation systems. A recent study by Haemmerli et al^13^ used a single point to evaluate the differences between augmented reality and pointer-based neuronavigation. Their results showed the superiority of augmented reality over the current pointer-based neuronavigation. Qi et al^14^ used a combination of marker-based system and printed tools to draw tumor boundaries which were compared across semiautomated MR systems with current neuronavigation. However, both studies did not demonstrate a system to verify surface matching of the holographic image. This would be prudent before MR can be used for more complex applications such as tumor biopsies, tumor resections, or placement of navigated external ventricular drains. Our study provides a validation method for establishing the degree of accuracy of MR systems that can be generalized across different software and hardware platforms. It eliminates the need for fiducials/marker-based navigation because this has largely been phased out with the newer navigation systems.

**FIGURE 6.**
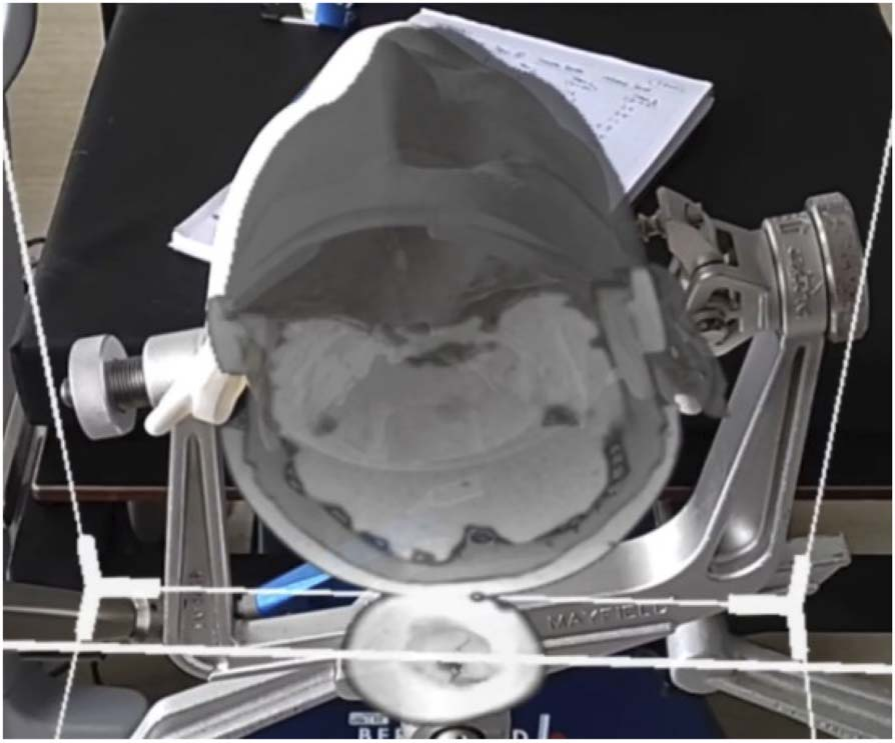
Superimposed MRI image being placed on the Realistic Operative Workstation for Educating Neurosurgical Apprentices model.

Our results have failed to demonstrate the equivalence of the MR-based navigation when compared with standard neuronavigation. As presented in Table, the magnitude of error of MR remains constantly below the acceptable accuracy of 5 mm and remains higher than that achieved by standard neuronavigation. This remains consistent across extracranial and intracranial surface landmarks, with the magnitude of error increasing for intracranial landmarks. We postulate multiple reasons for this. First, the software used in this study does not allow for autoregistration/autoplacement of the rendered image. The subjective placement of image on the model will contribute to the error magnitude obtained. Second, as shown in Figure 6, the contrast of the rendered image against the operating background will make it difficult for the user to accurately pinpoint bony landmarks. Nevertheless, despite the results being promising, significant work with the placement of rendered image will need to be performed before we can reach the current levels of accuracy available through conventional neuronavigation systems. A recent meta-analysis by Fick et al^15^ showed that there was lack of agreement regarding the best method to assess accuracy, but augmented reality systems reached a comparable accuracy (2.5 vs 2.6 mm).

Comparing the intracranial landmarks has shown interesting results. The magnitude of error increased for both standard neuronavigation and MR. The statistical significance was lost between the 2 groups. Although the small set of data points played a role in these results, it does show issues with navigation when patients are no longer in the supine position. The participants of this study provided verbal feedback in difficulties of ergonomics of registration using optical tracking when patients are placed in lateral position. Owing to the ease of manipulation of MR rendered image, the users found it simpler to place the image on the model without worrying about the placement of navigation tools/interference with other surgical drapes or devices around.

The authors collected subjective feedback from the participants of this study. As both users of the device had not used HoloLens before this, they believe that the learning curve was short. Within an hour after the session, the users felt comfortable in using the various tools provided by the hardware and software. Manipulation of image rendering because of unlimited degrees of freedom allowed the users to assess their placement in multiple coordinates without being restricted by the visibility of neuronavigation tools. They highlighted the need for autoregistration/autoplacement of the rendered image because that was the most time-consuming part of the registration and navigation process. The requirement for relatively high-speed internet to ensure smooth navigation of images would limit its use in poorly connected operating theaters.

The use of MR in medical applications is hugely promising but remains in its infancy. As we move beyond the proof-of-principle phase to that of a clinical tool, MR technology will have to achieve a high threshold of reliability and accuracy to supplant existing technology. This study is a first step in demonstrating the type of methodologies that can be used to achieve this.

### Limitations

This study does have the multiple limitations. The authors did not assess how accuracy varies for intracranial components. While the ROWENA model was scanned with its intracranial contents, the authors believe that the movement of the components would prevent in establishing the “ground truth” to compare across the 2 modalities. The authors did not assess how the accuracy would change once the patient is draped intraoperatively and if the degree of error increases in MR systems because of the lack of autoregistration and absence of checking any movement of the rendered image.

## CONCLUSION

As MR technology matures, there is an increasing need to validate its clinical utility formally. This study provides a relatively easy and feasible method to assess the accuracy of MR-based navigation without requiring any additions to the established imaging protocols. We failed to show the superiority of the MR-based navigation over the current neuronavigation systems. Further work will need to be performed before MR-based navigation systems can be used primarily for neuronavigation, and we propose that future studies of MR-based navigation provide data on navigation accuracy against both defined anatomic landmarks and established proprietary technology.
